# Bariatric surgery for non-alcoholic fatty liver disease in individuals with obesity (Base-NAFLD): protocol of a prospective multicenter observational follow-up study

**DOI:** 10.1186/s12893-021-01296-y

**Published:** 2021-06-24

**Authors:** Luyang Wei, Mengyi Li, Na Zeng, Yang Liu, Rixing Bai, Nengwei Zhang, Jinghai Song, Pin Zhang, Qiyuan Yao, Zhenghan Yang, Xinyan Zhao, Yun Zhang, Peng Zhang, Zhongtao Zhang

**Affiliations:** 1 Department of General Surgery, Beijing Friendship Hospital, Capital Medical University and National Clinical Research Center for Digestive Diseases, No. 95 Yong-an Road, Xi-Cheng, Beijing, 100050 China; 2 National Clinical Research Center for Digestive Diseases, Beijing Friendship Hospital, Capital Medical University, Beijing, 100050 China; 3 Department of General Surgery, Tiantan Hospital, Capital Medical University, No. 119, South West Ring Road, Fengtai, Beijing, 100070 China; 4 Department of General Surgery, Beijing Shijitan Hospital, Capital Medical University/Peking University, Ninth Clinical Medical College, Tieyilu 10, Yangfangdian, Haidian, Beijing, 100038 China; 5 Department of General Surgery, Beijing Hospital, National Center of Gerontology, No. 1 Dahua Road, Dong Dan, Dong-Cheng, Beijing, 100730 China; 6 Department of Bariatric and Metabolic Surgery, Shanghai Jiao Tong University Affiliated Sixth People’s Hospital, No.600 Yishan Road, Shanghai, 200233 China; 7 Center for Obesity and Metabolic Surgery, Huashan Hospital, Fudan University, No. 12 Middle Wulumuqi Road, Shanghai, 200040 China; 8 Department of Radiology, Beijing Friendship Hospital, Capital Medical University, No.95 Yong-an Road, Xi-Cheng, Beijing, 100050 China; 9 Liver Research Cennter, Beijing Friendship Hospital, Capital Medical University, No.95 Yong-an Road, Xi-Cheng, Beijing, 100050 China; 10 Department of Biobank, Beijing Friendship Hospital, Capital Medical University, No.95 Yong-an Road, Xi-Cheng, Beijing, 100050 China

**Keywords:** Bariatric surgery, Non-alcoholic fatty liver disease, Metabolic associated fatty liver disease, Sleeve gastrostomy, Roux-en-Y gastric bypass, One anastomosis gastric bypass

## Abstract

**Background:**

Bariatric surgery may be indicated in patients with nonalcoholic fatty liver disease (NAFLD) to achieve and maintain the degree of weight loss required to ensure therapeutic effects. However, bariatric surgery is still underrecognized in the treatment of NAFLD, including its inflammatory subtype, nonalcoholic steatohepatitis (NASH). Moreover, there is a lack of follow-up outcome data on different types of bariatric surgery in patients with NAFLD. This study aims to adequately assess the effect of bariatric surgery on NAFLD remission in obese patients.

**Methods:**

This prospective multicentre observational follow-up study will include 142 obese patients with NAFLD scheduled to undergo one of the following surgical procedures: sleeve gastrostomy, Roux-en-Y gastric bypass, and one anastomosis gastric bypass. The primary outcome is the complete remission rate of NAFLD one year postoperatively, which is defined by liver fat fraction < 5% on magnetic resonance imaging; the secondary outcomes includes (i) changes in NASH and liver fibrosis biopsy findings, (ii) changes in body weight and abdominal adipose weight, (iii) resolution of obesity-related comorbidities, and (iv) incidence of adverse events. A long-term follow-up related to this study will also be conducted.

**Discussion:**

This study will provide a necessary and preliminary foundation for the early identification and targeted treatment of patients with NAFLD who can be referred for bariatric surgery, as indicated for management of obesity and metabolic disease.

*Trial registration:* Clinicaltrials.gov: NCT04366999. Registered 21 April 2020. (https://clinicaltrials.gov/ct2/show/NCT04366999).

## Background

Obesity and obesity-related metabolic diseases have become a global public health crisis. Non-alcoholic fatty liver disease (NAFLD) is one of the most common comorbidities in obese patients, with a prevalence of approximately 25% worldwide [1]. NAFLD is a major cause of end-stage liver diseases, ranging from non-alcoholic simple hepatic steatosis and non-alcoholic steatohepatitis (NASH) to fibrosis and hepatic cirrhosis [1, 2]. NASH is estimated to become the leading cause of liver transplantation in the next ten years, which will result in immense economic and health burden worldwide [3]. High-quality evidence has demonstrated that obesity is the main risk factor for NAFLD [1]. The overall prevalence of obesity among patients with NAFLD and NASH was reported to be 51.34 and 81.83%, respectively [1, 4]. Particularly, more than 90% of severely obese patients undergoing bariatric surgery have NAFLD [5]. Presently, the treatment for patients with NAFLD includes thiazolidinediones, vitamin E supplementation, etc. However, pharmacotherapy has limited clinical application. The best data currently available indicate that overall weight loss is the key to alleviate NAFLD progression [1, 6, 7]. Bariatric surgery may be indicated in obese patients with NAFLD to achieve and maintain the degree of weight loss required to ensure therapeutic effects. However, bariatric surgery is still underrecognized in the treatment of NAFLD, including NASH, its inflammatory subtype. Furthermore, due to the controversies associated with the existing reports, randomised control trials (RCTs) comparing different types of surgery for NAFLD and their outcomes are urgently needed [8–12]. Currently, obesity with NAFLD is not a definite indication for bariatric surgery worldwide, according to the current clinical guidelines. Therefore, an observational follow-up study is crucial to create and assess novel RCT designs.

“Bariatric surgery for non-alcoholic Fatty Liver Diseases in Individuals with Obesity (Base-NAFLD)” is a prospective multicentre observational follow-up study. This study aims to adequately assess the effect of bariatric surgery on NAFLD remission in obese patients: (i) to demonstrate the effectiveness of bariatric surgery for NAFLD, including NASH, (ii) to explore the differences in the effectiveness among three types of surgery—sleeve gastrostomy (SG), Roux-en-Y gastric bypass (RYGB), and one anastomosis gastric bypass (OAGB), and (iii) to develop a predictive model for surgical treatment using internal and external validation cohorts. This study will provide a necessary and preliminary foundation for the early identification and targeted treatment of patients with NAFLD, who need to be referred for bariatric surgery for the management of obesity and metabolic diseases.

## Methods

### Patient recruitment

The Base-NAFLD study is a prospective, multicentre, observational study. Patients scheduled to undergo one of the three types of bariatric surgery—SG, RYGB, and OAGB—at the participating centres, between 21 April and 31 December 2020, will be screened for consecutive enrolment in this study. The corresponding follow-ups will be conducted 2 years postoperatively until December 2022.

### Types of surgery

Surgical procedures include SG, RYGB, and OAGB. SG is the most common procedure, followed by RYGB. OAGB has been recognised by The International Federation for the Surgery of Obesity and Metabolic Disorders(IFSO)since 2018 [13, 14].

### Inclusion criteria

Patients meeting the following inclusion criteria are being considered for enrolment:

1. Age between 16 and 65 years (all sexes).
2. Diagnosed with obesity according to the World Health Organization criteria for obesity in Asian populations [15] and scheduled for a primary bariatric surgery at the participating centres.
3. Diagnosed with hepatic steatosis preoperatively by radiologic (including ultrasonography, magnetic resonance imaging [MRI]-derived proton density fat fraction [PDFF]) or pathologic(intraoperative hepatic pathology) examinations.

### Exclusion criteria

Patients will be excluded if they meet any of the following exclusion criteria:

1. History of bariatric surgery or extensive gastrointestinal surgical procedures.
2. History of excessive alcohol consumption, defined as ethanol amounts converted from alcohol intake by men and women > 40 g/d and 20 g/d, respectively, for more than 5 years, or alcohol consumption > 80 g/d within 2 weeks (alcohol intake [g/d] = alcohol consumption [mL/d] × alcohol content [%] × 0.8).
3. History of taking amiodarone, methotrexate, tamoxifen, and glucocorticoids, or chronic use of any other drug that may induce liver injury.
4. Diagnosed with specific diseases that cause adiposis hepatica, including infections by hepatitis C virus genotype 3, hepatolenticular degeneration, autoimmune hepatitis, total parenteral nutrition, abetalipoproteinemia, congenital lipodystrophy, and chylous diarrhoea.
5. Diagnosed with confirmed or suspected malignant tumours or severe cardiorespiratory, cerebral, or metabolic diseases.
6. Pregnancy.
7. Not willing or unable to provide consent for participation in the study.

### Outcome measures

The primary outcome of this study is the complete remission rate of NAFLD one year postoperatively, as defined by liver fat fraction (LFF) < 5% on abdominal MRI.

Secondary outcomes are as follows:

(i) changes in NASH and liver fibrosis biopsy findings, including the NAS (NAFLD activity score) [16] and SAF scores (steatosis [S], activity [A], and fibrosis [F])[17] one year postoperatively.
(ii) changes in body weight and abdominal adipose weight, indicated by changes in the following parameters at each follow-up:
  - percent excess weight loss (%EWL): %EWL = ([initial weight] – [postoperative weight])/([initial weight] – [ideal weight]) (ideal weight is defined as a body mass index [BMI] of 25 kg/m^2^ at each follow-up point)
  - percentage of total weight loss (%TWL): %TWL = ([initial weight] – [postoperative weight])/([initial weight]) × 100
(III) resolution of obesity-related comorbidities: blood glucose levels, lipid levels, and liver enzyme levels.
(iv) incidence of adverse health events.

### Data collection

A standard case report form was designed before the study and transferred to an electronic data capture (EDC) system (https://www.base-nafld.com). The EDC system provides a graphical user interface for data entry and has a validation component for data cross-checking and a reporting tool for descriptive analysis. Well-trained clinical research coordinators from Chuankang (Medicine Technology Co, Beijing, China) and Suzhou Plenitude (Bio-Medical Technology Co., Ltd., Jiangsu, China) will retrieve all required information from the medical records of included patients and upload them into the EDC system. Clinical research associates from Beijing Funhau Medicine Technology Co. are in charge of monitoring and providing feedback on the EDC system and for data quality control.

The case report form was designed to collect the following baseline and follow-up data (Table 1):

1. Demographic data: study code, initial name, sex, date of birth, admission date, height, body weight, transumbilical abdominal circumference, smoking habits, and drinking habits.
2. Disease history and medication usage: type 2 diabetes mellitus, hypertension, polycystic ovarian syndrome, and obstructive sleep apnoea hypopnoea syndrome; use of oral hypoglycaemic, insulin, anti-hypertensive medication, and lipid-lowering drugs.
3. Preoperative examinations: routine blood tests and biochemical examinations, especially for ALT, AST, HbA1c, fasting blood glucose, fasting insulin levels, prothrombin activity time, and AFP; findings from abdominal ultrasonography and gastroscopy; and assessment of LFF levels from MRI.
4. Surgical information: operation date, type of surgery, operation time, and surgical procedure information, including gastric pouch volume, Roux limb length, biliopancreatic limb length, stapler usage, and suturing.
5. Liver pathology: findings from histopathological examinations of liver biopsy specimens obtained intraoperatively and one year postoperatively.
6. Adverse events or serious adverse events.

**Table 1:** Checklist of baseline and follow-up visits of patients enrolled in the Base-NAFLD study

	Visit 1 (Baseline)	Visit 2 (3-month intervals)	Visit 3 (6-month intervals)	Visit 4 (1-year intervals)	Visit 5 (2-year intervals)
Demographic data	●	●	●	●	●
Disease history and medication usage	●	●	●	●	●
Surgical information	●	✗	✗	✗	✗
Weight loss	✗	●	●	●	●
Routine blood tests	●	●	●	●	●
HbA1c	●	●	●	●	●
OGTT	●	✗	✗	○	✗
FBG	✗	●	○ ●	✗	●
Fasting insulin and fasting C-peptide	✗	○	○	●	○
Biochemical examination	●	●	●	●	●
Thyroid function	●	○	○	●	●
Serum iron and TIBC	●	●	○	●	●
Ferritin and folate and Vitamin B12	●	●	○	●	●
AFP	●	✗	✗	✗	●
Coagulation function	●	○	✗	●	●
Gastroscopy	●	✗	✗	○	○
C13 breath tests	●	✗	✗	○	○
Abdominal ultrasonography	●	●	○	○	○
Lower extremity vascular ultrasound	●	✗	✗	○	○
Abdominal MRI	●	●	●	●	●
Liver biopsy	●	✗	✗	●	✗
Adverse events	✗	●	●	●	●

Base-NAFLD, Bariatric Surgery for Non-alcoholic Fatty Liver Diseases in Individuals with Obesity; HbA1c, glycosylated haemoglobin; OGTT, oral glucose tolerance test; FBG, fasting blood glucose; TIBC, total iron-binding capacity; AFP, alpha-fetoprotein

●: Mandatory; ○: Optional; ✗: Not required

### Patient follow-up

According to the guidelines for the follow-up of patients undergoing bariatric surgery [18], follow-up visits at 3 months, 6 months, 1 year, and 2 years after surgery will be recommended to all patients. In each participating centre, follow-ups will be conducted by a research nurse or research doctor belonging to a multi-disciplinary team. Pertinent information will be obtained by either in-hospital visits, over telephone, or using WeChat—the most popular instant messaging app in China with over 1 billion users. Follow-up interviews have been designed to acquire information corresponding to the case report form (Table 1). Treatment-related adverse events and complications will be confirmed by all the researchers by a comprehensive review of involved medical records and details of follow-ups.

### Sample size determination

The Power Analysis and Sample Size software program (PASS, version 11.0 by NCSS, LLC) was used for sample size calculation, which was based on the estimated complete remission rate reported by previous retrospective studies [9–12] and the preliminary clinical results obtained from our centre. According to our preliminary data, 58 of the 69 patients (84%) who underwent bariatric surgery had preoperative underlying NAFLD, and the rate of complete remission was 55.2% three months postoperatively. To achieve a confidence interval width of 0.16 and an α value of 0.05 with a 78% complete remission rate, the estimated minimum sample size required for our study was 114. The withdrawal rate will be assumed to be 20% during follow-up; therefore, the sample size needed for this study will be at least 142 patients. Nevertheless, as this is an observational study, additional patients will be recruited for a subgroup analysis. According to the national registry in our country, the number of patients undergoing each type of surgery was found to be 82, 30, and 30 patients for the SG, RYGB, and OAGB groups, respectively.

### Statistical analysis

SPSS 20.0 (SPSS, Inc., Chicago, IL, USA) and R software (R version 3.6.3; R Foundation for Statistical Computing, Vienna, Austria) will be used to perform statistical analyses. Continuous data will be presented as mean (standard deviation) or median (minimum, maximum) values, and categorical data will be presented as numbers (proportions/frequencies/percentages). P-values < 0.05 will be considered statistically significant.

1. Comparison between preoperative and postoperative parameters: For paired continuous data, the paired t-test (normally distributed variables) or Wilcoxon’s signed-rank (skewed variables) will be used for analysis, whereas for paired categorical data, the chi-square test or Fisher’s exact test will be used for analysis.
2. Primary outcomes: The complete remission rate of NAFLD in obese patients, along with the corresponding 95% confidence interval will be reported.
3. Sensitivity analysis: A sensitivity analysis will be conducted to investigate the differences between the three types of surgery and BMI subgroups in terms of complete remission rate of NAFLD. Continuous variables will be tested by a paired t-test or Wilcoxon’s signed-rank test for paired samples, and the association between covariates and main outcomes will be tested using the chi-square test or Fisher’s exact test.
4. Development and validation of the prediction model: Before building the model, the dataset will be divided into a training set—three-quarters of the enrolled patients will be randomly assigned to this set—and a validation set—the remainder of the patients will be assigned to this set. A multivariable logistic regression model will be used in which the response variable will be complete remission of NAFLD. The model will be built using stepwise selection based on the Akaike information criterion. The training model will be subsequently applied to the validation set. The model performance of both the training and validation sets will be evaluated using C statistics for discrimination, and the goodness-of-fit will be evaluated using Hosmer–Lemeshow test. A calibration curve will also be developed to examine the model fit. For both the training and validation sets, the predicted risk will be plotted against the observed risk for each of the 10 risk percentiles created from the dataset. Calibration will be evaluated using the Hosmer–Lemeshow goodness-of-fit test and chi-square test based on the predicted risk deciles. Results will be displayed as a nomogram to provide clinicians with an intuitive and quantitative tool to predict the probability of complete NAFLD remission in obese patients.

### Quality control

Standard operating procedures, MRI fat quantification sequences, PDFF measurements, liver biopsy, hepatic pathology report, biobank, follow-up visits, and source data collection will be well documented after discussing with the relevant specialists in the multidisciplinary team. Prior to this study, all participants and study personnel, including primary investigators, sub-primary investigators, research doctors, nurses, clinical research coordinators, and clinical research associates, will receive adequate training. Clinical research associates will check and verify the authenticity, accuracy, and integrity of all information based on the source data. Data modification traces will be recorded in the EDC system. After verification, the data will be locked for the final statistical analysis. Furthermore, the research committee will hold regular meetings to discuss issues concerning the progress and quality control of this study, which is under the supervision of the Beijing Municipal Health Commission.

## Discussion

Given the current understanding of the pathogenesis of NAFLD and its rising prevalence, an international expert consensus was reached, highlighting the need for a new definition for NAFLD as metabolic associated fatty liver disease (MAFLD) [19]. Obesity is one of the main criteria for MAFLD based on evidence indicating hepatic steatosis. Due to the lack of approved pharmacotherapies, bariatric surgery has the potential to ameliorate NAFLD in patients with obesity. According to the current literature, bariatric surgery significantly improves biochemical and histologic parameters in patients with NAFLD [8]. However, most studies on this matter are retrospective, single-centre studies that considered laboratory values as assessment measures, with limited ability to draw definitive conclusions [9, 10]. The primary outcome in our study is the complete remission rate of NAFLD at 1 year after bariatric surgery, as measured by MRI. Moreover, the changes in the liver biopsy findings pertaining to NAFLD activity and fibrosis staging scores will be calculated as important secondary outcomes.

NAFLD is diagnosed using several methods, including imaging, blood biomarkers/scores, or liver biopsy. Liver biopsy is deemed as the ‘golden standard’ method in patients without any history of alcohol or liver toxin exposure. As a surrogate for invasive measurement, laboratory values can assess NAFLD and liver fibrosis [20]. However, biochemical values, such as ALT and AST, cannot accurately and quantitatively evaluate hepatic steatosis, and the influence of liver injury on liver enzyme levels cannot be excluded [21]. Ultrasonography is a widely used first-line diagnostic modality but has limited sensitivity. It does not reliably detect steatosis of < 20%, and its performance is suboptimal in individuals with a BMI > 40 kg/m^2^ [19, 21]. Furthermore, computed tomography has limited ability in quantifying hepatic fat fraction [22]. MRI, as a non-invasive diagnostic modality, is an accepted reference imaging method and an alternative to liver biopsy for hepatic fat content assessment [22]. Moreover, PDFF calculated by MRI accurately estimates the presence of hepatic steatosis [23, 24]. Significant decreases in LFF features are visible on MRI after bariatric surgery [25].

Many studies have shown that bariatric surgery significantly ameliorates NAFLD in obese patients [26]. Particularly, Chaim et al. used clinicopathologic findings to confirm that bariatric surgery can effectively alleviate NAFLD [27]. However, there is still no consensus on the optimal choice of surgical procedure for NAFLD. According to an Indian prospective study, the remission rate in the SG group was higher than that in the RYGB group [9]. However, Yeo et al. used NAFLD fibrosis scores to compare the degree of liver fibrosis remission one year postoperatively and showed that patients who underwent RYGB had better outcomes than those who underwent SG [10]. Kalinowski et al. performed a secondary analysis of an RCT and compared surgical outcomes between RYGB and SG groups at 12 months postoperatively; they found no significant differences in AST and ALT levels between both the groups [11]. Similarly, Lee et al. evaluated 5741 obese patients with clinical NASH scores and found that RYGB, SG, and OAGB all have significant and lasting ameliorative effects on NASH [12]. Moreover, Jimenez et al. compared the NAFLD fibrosis score between the period before surgery and at 12, 24, and 36 months after RYGB, considering the fact that the post-surgical weight regained after RYGB may partly mitigate NAFLD improvement; however, further research is needed on this matter [28]. Most of the aforementioned studies were retrospective, conducted in a single centre, and had an observational design with short-term follow-ups. High-quality evidence from studies with a sufficiently large sample size is needed to demonstrate the effectiveness of bariatric surgery for NAFLD and explore any differences in effectiveness between the standard types of surgery.

As this is a multicentre observational follow-up study, we selected tertiary hospitals with extensive clinical and research experience with bariatric surgery to overcome the limitations of surgical procedure bias and inadequate data recording and ensure a high-quality research process. Moreover, essential standard operating procedures were peer-reviewed by all researchers before the study began. Researchers with a Good Clinical Practice certificate issued by the China Food and Drug Administration received systematic training to ensure adherence to the study protocol. Third party monitoring and coordinate investigators meetings will be conducted regularly throughout the study to ensure the authenticity and integrity of data.

To the best of our knowledge, this is the first prospective multicentre observational follow-up study that will adequately assess the usefulness of bariatric surgery, including three mainstream surgical procedures, on NAFLD remission in patients. By exploring the primary and secondary outcomes, the risk factors that strongly contribute to complete NAFLD remission will be identified to develop and verify the predictive model. This study will also provide preliminary results on the optimal choice of surgery type for patients with NAFLD as the presence of NAFLD in obese patients is not a definite indication for bariatric surgery worldwide according to the current clinical guidelines. Moreover, by investigating the preoperative factors associated with complications, we will be able to identify modifiable factors to prevent complications in the future. In summary, this study will improve our understanding of NAFLD development in obese patients and identify the factors influencing its outcomes, facilitating the optimisation of individualised treatment and eventually providing high-quality evidence for the indication of bariatric surgery for NAFLD.

## Data Availability

The datasets generated and/or analysed during the current study are updated in a national registry named “Greater China Metabolic and Bariatric Surgery Database (GC-MBD)” (Clinicaltrial.gov: NCT03800160) repository, which are available from the corresponding author on reasonable request. Protocol available at ClinicalTrials.gov (ClinicalTrials.gov Identifier: NCT04366999).

## Abbreviations

NAFLD: Non-alcoholic fatty liver disease
NASH: Non-alcoholic steatohepatitis
MAFLD: Metabolic associated fatty liver disease
RCT: Randomised control trial
BMI: Body Mass Index
TWL: Total weight loss
EWL: Excess weight loss
SG: Sleeve gastrostomy
RYGB: Roux-en-Y gastric bypass
OAGB: One anastomosis gastric bypass
MRI: Magnetic resonance imaging
PDFF: Proton density fat fraction
LFF: Liver fat fraction
NAS: NAFLD activity score
HbA1c: Glycosylated haemoglobin
ALT: Alanine transaminase
AST: Aspartate transaminase
AFP: Alpha-fetoprotein
EDC: Electronic data capture
